# Semaglutide in MASLD Patients: Improved Survival and Liver Outcomes

**DOI:** 10.3390/ph18071075

**Published:** 2025-07-21

**Authors:** Mohamad Suki, Johnny Amer, Yael Milgrom, Muhammad Massarwa, Wadi Hazou, Yariv Tiram, Ofer Perzon, Yousra Sharif, Joseph Sackran, Revital Alon, Nachum Emil Eliezer Lourie, Itamar Raz, Ashraf Imam, Abed Khalaileh, Rifaat Safadi

**Affiliations:** 1 Institution of Liver Diseases, Hadassah Medical Organization, Hadassah-Hebrew University, Jerusalem 91120, Israelsafadi@hadassah.org.il (R.S.); 2 Transplantation Unit, Department of Surgery, Hadassah Medical Organization, Hadassah-Hebrew University, Jerusalem 91120, Israel

**Keywords:** semaglutide, MASLD, liver outcomes, cirrhosis, mortality

## Abstract

**Introduction**: Semaglutide (SEMA) has shown potential benefits in metabolic dysfunction-associated steatotic liver disease (MASLD). This large real-world study aimed to evaluate the effects of SEMA on MASLD patients’ clinical outcomes and liver-related complications. **Results**: Following propensity score matching based on 34 variables (demographics, comorbidities, laboratory tests, and medication history), SEMA-treated (*n* = 19,112) patients were compared with non-SEMA (*n* = 19,112) cases. Both cohorts were well-balanced, except for higher BMI in the SEMA group (36.60 ± 6.25 vs. 34.89 ± 6.84 kg/m^2^). After one year, the SEMA group demonstrated ~one BMI point reduction but maintained significantly higher BMI (35.51 ± 6.34 vs. 34.11 ± 6.64, *p* < 0.001). LDL, triglycerides, and HbA1c levels significantly improved with SEMA, as evidenced by decreased rates of poor metabolic markers (31.13% vs. 34.32%, *p* < 0.001). The SEMA-treated patients demonstrated significantly higher survival, lower cardiovascular risk, and reduced progression to advanced liver disease compared to controls. **Discussion**: In this large real-world cohort, SEMA use in MASLD patients was associated with significantly improved 1-year survival, cardiovascular, and liver-related outcomes. These benefits appear to result primarily from metabolic improvements and anti-inflammatory effects. **Materials and Methods**: Data were sourced from TriNetX, a global health research platform with de-identified electronic medical records spanning 135 million patients across 112 healthcare organizations worldwide. We included MASLD adults diagnosed according to ICD9 criteria. Assessed outcomes included survival, biochemical, hematologic, AFP, metabolic and cardiovascular parameters, advanced liver disease (ALD), synthetic function, and metabolic markers. **Conclusions**: Semaglutide may serve as an effective therapeutic strategy to improve outcomes in MASLD.

## 1. Introduction

Metabolic dysfunction-associated steatotic liver disease (MASLD), previously known as non-alcoholic fatty liver disease (NAFLD), represents a significant global health burden, affecting approximately 25–30% of the world’s population and increasingly becoming the leading cause of chronic liver disease worldwide [1,2]. MASLD encompasses a spectrum of conditions ranging from simple steatosis to metabolic dysfunction-associated steatohepatitis (MASH), previously known as non-alcoholic steatohepatitis (NASH). MASH can progress to advanced fibrosis, cirrhosis, and hepatocellular carcinoma (HCC) [3]. The disease is closely linked to metabolic syndrome components, including obesity, type 2 diabetes mellitus (T2DM), dyslipidemia, and hypertension, which collectively contribute to its pathogenesis and progression [4,5].

The global prevalence of MASLD continues to rise in parallel with the obesity epidemic, with more than 250 million individuals affected globally and the number of individuals in advanced stages expected to double by 2030 [6,7]. Among people with overweight or obesity, approximately one-third also live with MASLD, significantly impacting their health and representing a substantial unmet clinical need [8]. The risk of disease progression to advanced liver disease, including liver cancer, is higher in MASLD patients compared to the general population, highlighting the importance of early intervention [9].

Despite its high prevalence and potential for serious complications, therapeutic options for MASLD remain limited. Current management strategies primarily focus on lifestyle modifications, including weight loss through diet and exercise, which have shown efficacy but are often difficult to maintain long-term [10]. While several pharmacotherapies have been investigated, including vitamin E, pioglitazone, and obeticholic acid, until recently, none had received regulatory approval specifically for MASLD treatment, highlighting an urgent need for effective interventions [11]. In March 2024, the U.S. Food and Drug Administration (FDA) granted accelerated approval to resmetirom, a thyroid hormone receptor beta agonist, making it the first medication specifically approved for treating noncirrhotic MASH with moderate to advanced liver fibrosis [12,13].

Glucagon-like peptide-1 receptor agonists (GLP-1 RAs), initially developed for T2DM management, have emerged as promising candidates for MASLD treatment due to their metabolic and weight-reducing effects [14]. Semaglutide, a long-acting GLP-1 RA with established efficacy in glycemic control and weight reduction, has garnered particular interest in this context [15]. Beyond its metabolic benefits, semaglutide exhibits pleiotropic effects, including anti-inflammatory and potential antifibrotic properties, which may be especially beneficial in liver disease [16,17]. Notably, recent clinical data indicate that semaglutide may have beneficial effects on liver histology, including a reduction in liver fat content and improvement in markers of inflammation [18].

The landmark phase 2 trial of semaglutide in NASH demonstrated that once-daily subcutaneous semaglutide significantly improved NASH resolution without worsening fibrosis compared to placebo [19]. Additionally, the STEP program has demonstrated substantial weight loss with once-weekly semaglutide, an effect that could indirectly benefit MASLD patients [10]. More recently, the phase 3 ESSENCE trial demonstrated that semaglutide 2.4 mg significantly improved liver fibrosis and resolved MASH compared to placebo in patients with fibrosis stage 2 or 3, positioning semaglutide as a potential future therapy for this indication [20,21]. However, clinical trials were conducted in carefully selected patient populations under controlled conditions, potentially limiting their generalizability to real-world clinical practice.

Real-world evidence is crucial in supplementing clinical trial data, particularly for complex conditions like MASLD, where patient heterogeneity and comorbidities can significantly impact treatment outcomes. Real-world studies offer insights into medication effectiveness and safety in diverse patient populations that better represent everyday clinical practice [22]. Despite semaglutide’s promising profile, comprehensive real-world data on its effects in MASLD patients remain scarce, particularly regarding long-term outcomes such as mortality, liver-related complications, and cardiovascular events.

The present study aimed to evaluate the effect of semaglutide therapy on liver-related outcomes and overall survival in patients with MASLD in a real-world setting.

## 2. Results

### 2.1. Baseline Characteristics

The matched cohort included 19,112 semaglutide-exposed and 19,112 semaglutide-unexposed MASLD patients. Baseline characteristics are presented in Table 1a–c, covering demographics, comorbidities, laboratory parameters, and medication use. Rigorous propensity score matching across 34 variables minimized confounding, ensuring comparability between groups. Some statistically balanced residual differences persisted, but combinations of *p*-values and standardized mean differences confirmed adequate overall between-group balancing to enable the isolated assessment of semaglutide treatment effects.

#### 2.1.1. Demographics (Table 1a)

The two cohorts were well-balanced for key demographic and clinical characteristics, with the only notable difference being higher baseline BMI in the semaglutide group (Table 1a).

#### 2.1.2. Comorbidities (Table 1a)

The prevalence of key metabolic comorbidities, including diabetes, hypertension, and cardiovascular disease, was well-balanced between groups (Table 1a).

#### 2.1.3. Laboratory Parameters (Table 1b)

Baseline laboratory values revealed modest but statistically significant differences, with lower liver enzymes, improved markers of liver synthetic function (bilirubin, albumin, platelet count, INR), and slightly better metabolic profiles in the semaglutide group. Despite these differences, standardized mean differences remained within acceptable limits, indicating well-balanced cohorts (Table 1b).

#### 2.1.4. Medications (Table 1c)

Medication use prior to the index date, including glucose- and lipid-lowering therapies, was comparable between groups, with minor differences unlikely to impact outcomes (Table 1c). Overall, medication matching ensured that any observed differences in outcomes could be more confidently attributed to semaglutide rather than other pharmacological interventions.

### 2.2. Clinical Outcomes

Table 2a–c summarize the central results regarding the impact of semaglutide exposure on major clinical outcomes (Table 2a), absolute laboratory data (Table 2b), and categorical laboratory data according to defined cutoffs (Table 2c).

#### 2.2.1. Mortality and Survival (Table 2a, Figure 1)

**Figure 1:**
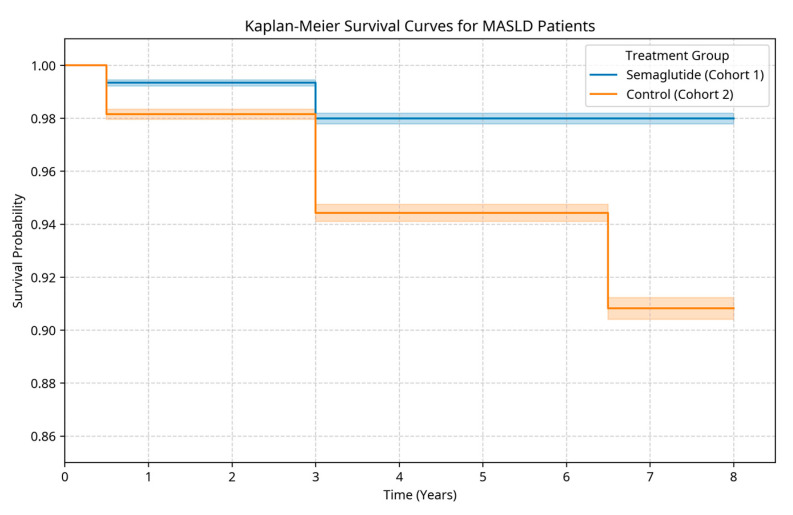
Kaplan–Meier survival curves. Legend: This figure presents the Kaplan–Meier survival analysis comparing the long-term survival of propensity score-matched MASLD patients with and without semaglutide exposure over an 8-year follow-up period. The blue line represents semaglutide-treated patients; the red line represents controls. The width of each line represents the standard deviation. The x-axis represents the survival time in days after the index event, and the y-axis represents the proportion of surviving patients.

Semaglutide treatment was associated with significantly improved long-term survival, as demonstrated by Kaplan–Meier analysis and a 63% relative risk reduction in all-cause mortality (Figure 1, Table 2a).

**Figure 2:**
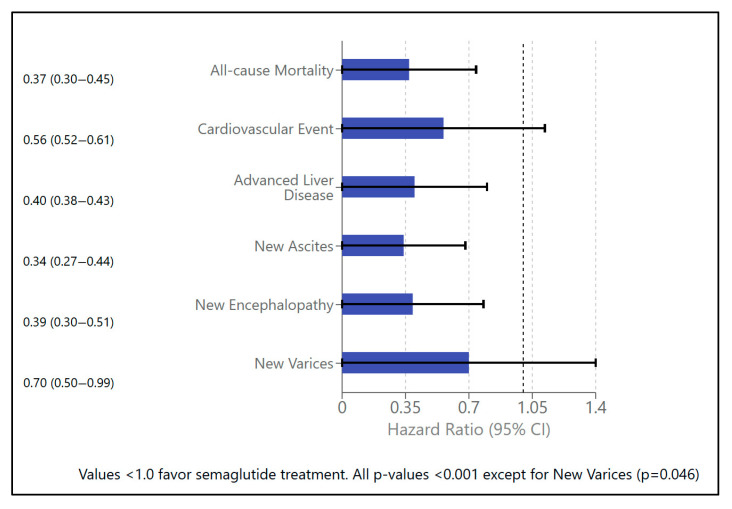
Forest plot of hazard ratios (semaglutide vs. non-semaglutide).

The calculated hazard ratio for all-cause mortality was 0.37 (95% CI 0.30–0.45), indicating a 63% reduced risk of death in the semaglutide group compared to the control group over the entire follow-up period (Figure 2). The number needed to treat (NNT) to prevent one death decreased with longer follow-up, reflecting the cumulative benefit of semaglutide therapy over time (Table 3).

#### 2.2.2. Metabolic Profile (Table 2a,b)

Semaglutide therapy was associated with durable improvements in metabolic profiles, including BMI, glycemic control, and lipid parameters. These effects were maintained across all time points and align with the expected benefits of GLP-1 receptor agonists. These findings demonstrate semaglutide’s sustained beneficial effects on metabolic health throughout the follow-up period.

#### 2.2.3. Cardiovascular Events (Table 2a)

Cardiovascular outcomes were consistently better in the semaglutide group, with a significantly lower incidence of major events throughout follow-up (Table 2a and Figure 2), translating into a 44% relative risk reduction. The NNT to prevent one cardiovascular event was favorable across all time points (Table 3).

#### 2.2.4. Liver Function Parameters (Table 2b,c, Figure 3)

**Figure 3:**
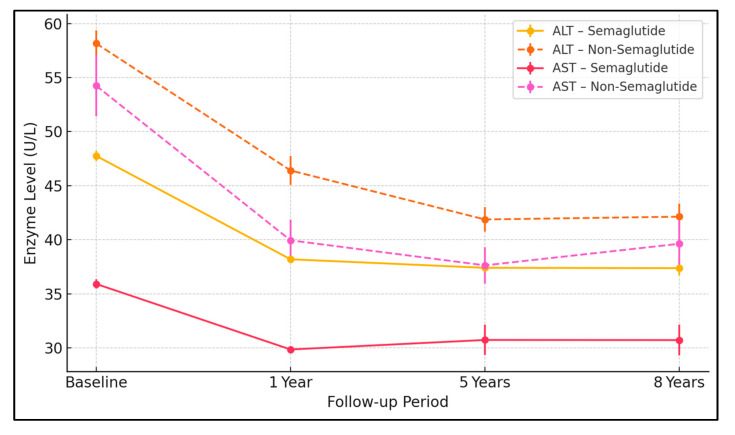
Liver enzyme trends over time. Mean ALT and AST values ± standard errors at 1, 5, and 8 years of follow-up are presented by continuous lines in the SEMA group and dashed lines in the non-SEMA group. The color key is illustrated. All differences were significant at each time point (*p* < 0.001). The semaglutide group maintained lower liver enzymes throughout the follow-up.

Liver enzymes and markers of hepatic synthetic function improved significantly in the semaglutide group, suggesting reduced hepatic inflammation and preserved liver function. Detailed trends are presented in Table 2b,c as well as Figure 3.

#### 2.2.5. Advanced Liver Disease (Table 2a–c)

**Figure 4:**
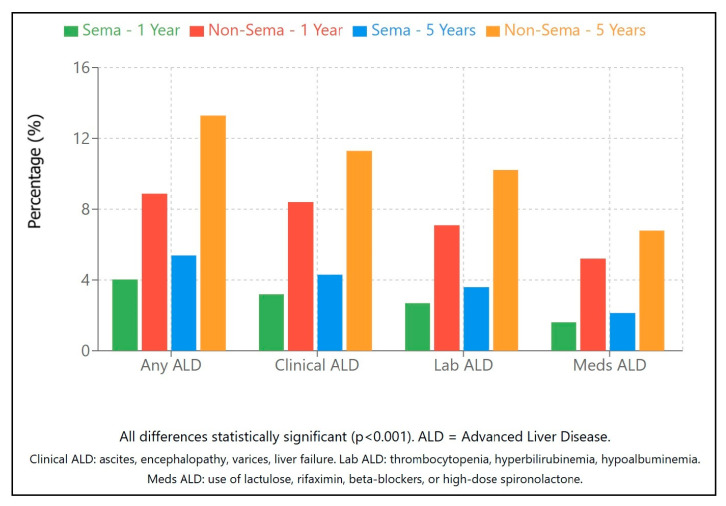
Advanced liver disease outcomes. Outcomes are illustrated as percentages (%).

The risk of developing advanced liver disease was markedly lower among semaglutide users, with consistent reductions across clinical, laboratory, and treatment-based definitions (Figure 4). These findings underscore semaglutide’s potential hepatoprotective effects.

#### 2.2.6. Other Outcomes and Subgroup Analyses (Table 2a)

The incidence of cirrhosis-related complications (e.g., ascites, encephalopathy, varices) and the need for liver-specific medications were lower with semaglutide, further supporting its protective effects against hepatic decompensation.

## 3. Discussion

This large real-world retrospective cohort of MASLD patients, followed for up to 8 years, demonstrates that semaglutide therapy is associated with significantly improved overall survival and reduced liver-related complications. The rigorous propensity score matching of 34 parameters overcomes selection bias and allows a robust comparison between semaglutide-exposed and unexposed groups, minimizing confounding factors related to demographics, comorbidities, liver disease severity, and metabolic status at baseline. The absolute mortality reduction of 1.11% at 1 year and 2.39% at 5 years represents a substantial clinical benefit, particularly considering that these were predominantly ambulatory patients without advanced liver disease at baseline. The magnitude of this survival benefit is consistent with or exceeds that observed in clinical trials of interventions for metabolic diseases [23], highlighting semaglutide’s potential role in improving long-term outcomes in MASLD patients.

Our findings regarding metabolic parameters and cardiovascular outcomes align with previous studies of GLP-1 RAs [23,24]. Despite starting with a higher baseline BMI, semaglutide-treated patients achieved modest but significant weight loss and improved lipid and glycemic profiles. Cardiovascular outcome trials like SUSTAIN-6 [18] and PIONEER 6 [19] showed cardiovascular benefits of semaglutide in diabetic populations, while other GLP-1 RAs like liraglutide [15] also demonstrated CV benefits. Our real-world data complement these findings by demonstrating a 44% relative risk reduction in cardiovascular events across all follow-up periods in this MASLD cohort.

The comprehensive reduction in ALD risk across multiple classification methods (Figure 4) provides particular confidence in semaglutide’s hepatoprotective effects. The similar risk reductions observed in clinically defined ALD, laboratory-defined ALD (HR 0.36), and ALD requiring medications (HR 0.32) suggest that semaglutide’s benefits extend beyond subjective clinical assessments to include objective laboratory parameters and treatment needs. This consistency across different ALD definitions strengthens the evidence for semaglutide’s efficacy and suggests multiple complementary mechanisms through which the drug may exert its hepatoprotective effects, including reducing inflammation (reflected in clinical presentations) [20], improving liver function (reflected in laboratory parameters), and decreasing the need for ALD-specific medications.

Perhaps most striking are the liver-specific benefits observed with semaglutide. The greater than 50% relative risk reduction in progression to advanced liver disease might change the natural history of MASLD [24]. This far exceeds the effects typically observed with lifestyle modifications alone and is comparable to or better than results reported with other pharmacological interventions such as cenicriviroc (which failed to show efficacy on fibrosis in phase 3 despite phase 2b promise [25]) or obeticholic acid [8] and potentially complementary to the effects seen with bariatric surgery [26]. The superior outcomes in patients with higher BMI and those with diabetes are consistent with the proposed mechanisms of action and suggest potential target populations for semaglutide therapy.

The mechanisms underlying semaglutide’s hepatoprotective effects are likely multifactorial [24,27]. Direct anti-inflammatory [20] and antisteatotic effects have been demonstrated in preclinical models, where GLP-1 RAs reduced hepatic fat content, improved mitochondrial function, and attenuated oxidative stress [21]. The drug’s effects on weight loss [10,13] and insulin sensitivity [27] further contribute to reduced hepatic fat accumulation and inflammation. A recent mechanistic study suggests that GLP-1 RAs may also have direct effects on hepatic stellate cells, potentially reducing fibrogenesis. The consistent improvement in liver enzymes (particularly ALT and AST) observed in our study supports a direct anti-inflammatory effect [12]. Previous studies have shown that ALT normalization correlates with histological improvement in NASH and a reduced risk of disease progression [6]. The preservation of synthetic liver function in semaglutide-treated patients further indicates a substantial impact on disease course, as declining synthetic function typically heralds the onset of cirrhotic complications.

Our findings have important clinical implications as they suggest that semaglutide’s benefits in MASLD extend beyond improvements in surrogate markers (like those assessed via non-invasive tests [23]) to clinically meaningful outcomes, including reduced mortality and lower rates of advanced liver disease. The calculation of NNT values provides practical guidance for clinicians, showing that the benefits of semaglutide become more apparent with longer treatment duration. These real-world findings, alongside other similar efforts [28], support the ongoing clinical development and potential positioning of semaglutide in MASLD management strategies [29,30].

Our study has several notable strengths, including its large sample size (19,112 patients per arm), extensive propensity score matching, and long follow-up period. The real-world setting enhances the generalizability to clinical practice [14], where patients often have comorbidities and concomitant medications that would exclude them from clinical trials. The consistent findings across multiple outcome domains and time points provide robust evidence for semaglutide’s benefits in MASLD.

A key limitation of this study relates to the analytical constraints imposed by the TriNetX platform. Although propensity score matching was rigorously applied to balance baseline characteristics, we could not implement more sophisticated statistical models, such as multivariable logistic regression, to further adjust for potential confounding. Consequently, the observed associations, while compelling, must be interpreted with caution.

The large sample size of over 19,000 patients per group enhances the statistical power of our analysis and increases confidence in detecting clinically meaningful differences. Nevertheless, as this was a retrospective study, no formal prospective power calculation was performed.

As with all observational studies, residual confounding cannot be completely eliminated despite extensive matching [14]. The TriNetX database lacks detailed information on lifestyle factors such as dietary habits and physical activity, which could influence outcomes. Additionally, we did not have access to liver biopsy data to confirm NASH diagnosis or assess histological changes; non-invasive assessments like blood biomarkers or imaging scores [23] were also not systematically available. Finally, medication adherence could not be directly assessed, though the persistent metabolic effects observed suggest reasonable adherence in the semaglutide group.

## 4. Materials and Methods

### 4.1. Data Source

This retrospective cohort study utilized real-world data from the TriNetX global health research platform (TriNetX LIVE™, LLC, Cambridge, MA, USA) as of 30 December 2023. TriNetX includes de-identified longitudinal electronic health records from over 135 million patients across 112 healthcare organizations worldwide, encompassing hospitals, primary care clinics, and specialty centers. The database includes demographic information, diagnoses (ICD-9/10), procedures, medications (orders, prescriptions, and administrations), laboratory test results (LOINC codes), and healthcare utilization (Appendix A–C). The study involved the analysis of existing de-identified records but not the raw data, and therefore was exempt from Institutional Review Board approval.

### 4.2. Study Population and Cohort Definitions

**Figure 5:**
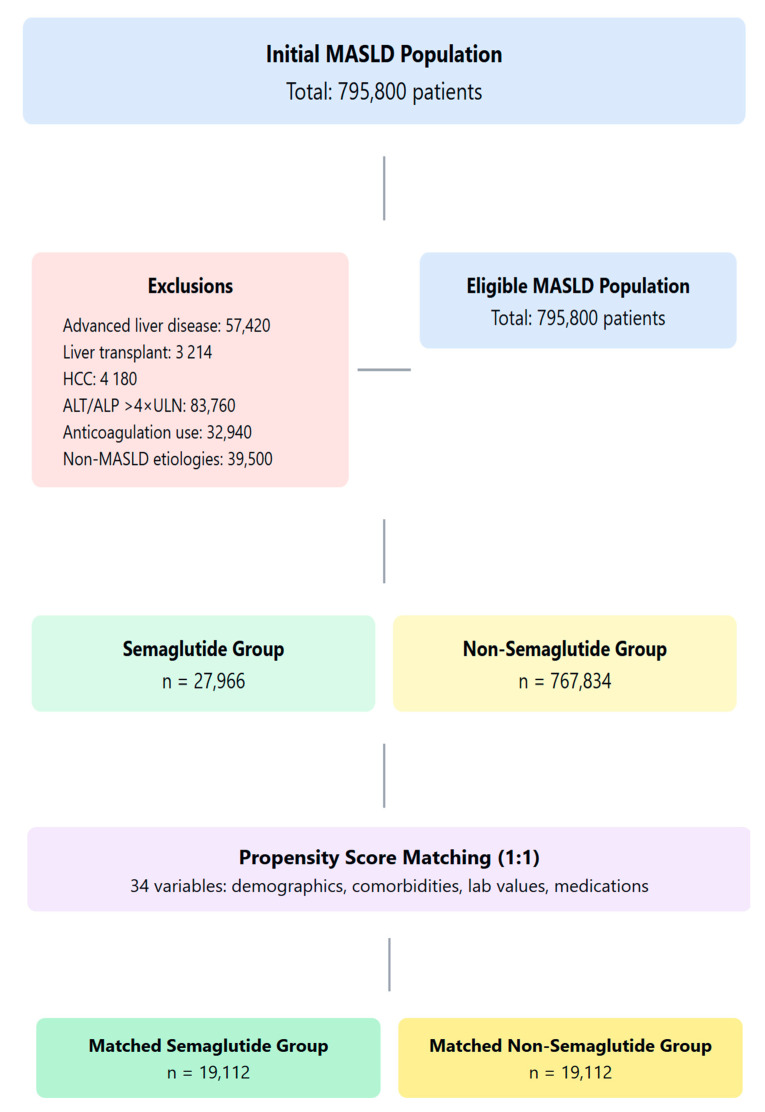
Study flow diagram.

Eligible patients were adults with metabolic dysfunction-associated steatotic liver disease (MASLD, MASH, NAFLD, NASH) diagnosed according to ICD-9 criteria. The study population included patients aged 18–90 years with established MASLD diagnosis who had adequate follow-up data. The age range was selected to represent adult MASLD populations while excluding extremes of age where MASLD phenotypes differ, consistent with prior large cohort studies [1]. The study flow is illustrated in Figure 5.

Two cohorts were defined based on semaglutide exposure:

**Semaglutide-exposed group**: patients prescribed semaglutide at or before the index date, with continued exposure throughout the follow-up period. All approved doses of semaglutide were included (0.25 mg, 0.5 mg, 1.0 mg, and 2.4 mg), administered via either subcutaneous injection or oral formulation.

**Semaglutide-unexposed group**: patients with no semaglutide prescriptions at baseline or during the follow-up period. This group received the standard of care for MASLD, which could include other antidiabetic, antihypertensive, or lipid-lowering medications.

### 4.3. Exclusion Criteria

Patients were excluded for any of the following: (1) ALT > 4×UNL or ALP > 4×UNL at or before the index date, to avoid confounding from acute liver injury or cholestatic disorders; (2) advanced liver disease, defined by diagnoses of cirrhosis or portal hypertension at baseline; (3) liver transplantation or hepatocellular carcinoma history, as these were study outcomes; (4) the use of anticoagulants, to preserve INR evaluation integrity; and (5) coexisting chronic liver diseases of non-metabolic etiology, including viral hepatitis, autoimmune hepatitis, primary biliary cholangitis, primary sclerosing cholangitis, Wilson’s disease, hemochromatosis, or alpha-1 antitrypsin deficiency.

Appendix A summarizes the cohort definitions and TriNetX codes of the inclusion and exclusion criteria.

### 4.4. Propensity Score Matching

Propensity scores were calculated using logistic regression incorporating 34 covariates, including demographics (age, gender, race), comorbidities (diabetes, hypertension, cardiovascular conditions), laboratory values (liver enzymes, metabolic parameters), and medications. These covariates were drawn from the 12 months preceding the index date to reflect a contemporaneous clinical profile.

A 1:1 greedy matching algorithm (without replacement) was applied using a standard caliper width, resulting in 19,112 matched pairs. Covariate balance was assessed using standardized mean differences (St. Diff.), with <0.3 considered acceptable. *p*-values were interpreted only when St. Diff. ≥ 0.3.

The use of propensity score matching for observational cohort studies is a well-established method to reduce confounding, as described by Austin [31] and Stuart [32].

### 4.5. Outcomes

Outcomes were assessed at 1, 5, and 8 years following the index date and included:

- Primary outcomes: all-cause mortality and overall survival
- Liver-related outcomes: advanced liver disease (ALD) was defined according to clinical diagnoses (e.g., portal hypertension, ascites, varices, hepatic encephalopathy), laboratory abnormalities (e.g., thrombocytopenia, hyperbilirubinemia, hypoalbuminemia, hyperammonemia), or dispensed medications used specifically for cirrhosis (e.g., propranolol, lactulose, rifaximin, spironolactone).
- Cardiovascular events: myocardial infarction, stroke, atrial fibrillation, and heart failure
- Metabolic parameters: LDL, HDL, triglycerides, HbA1c, BMI
- Disease progression: changes in liver enzymes, liver synthetic function, and the development of liver-related complications

Composite outcomes were created using a combination of clinical diagnoses, lab abnormalities, and relevant medication use. Detailed coding algorithms for outcome definitions are provided in Appendix B and Appendix C.

### 4.6. Statistical Analysis

Kaplan–Meier curves were used to evaluate survival and mortality over time. Hazard ratios (HRs) with 95% confidence intervals (CIs) were calculated using Cox proportional hazards models adjusted for relevant baseline variables. The number needed to treat (NNT) to prevent one clinical outcome was calculated as the reciprocal of the absolute risk reduction.

Continuous variables were compared using *t*-tests, and categorical variables using chi-square tests. Results are expressed as means ± standard deviations or proportions. As distributions were normal in all parameters, they are not shown. Correlation analyses were performed using Pearson’s correlation coefficient to assess the relationship between changes in BMI and liver enzyme levels. Subgroup analyses were conducted to identify the patient characteristics associated with differential treatment effects. A two-sided *p*-value < 0.05 was considered statistically significant.

The normality of continuous variables was assessed using a visual inspection of histograms and Shapiro–Wilk tests where applicable. Given the large sample size and central limit theorem assumptions, parametric tests were deemed appropriate.

It is important to acknowledge that our analysis was constrained by the inherent limitations of the TriNetX platform. While extensive propensity score matching was applied to minimize selection bias, we were unable to employ alternative statistical models such as logistic regression for outcome analysis due to platform restrictions. Therefore, despite our efforts to adjust for confounding, residual confounding and model limitations remain possible.

## 5. Conclusions

In conclusion, this long-term study provides suggestive evidence that semaglutide therapy confers durable benefits in patients with metabolic dysfunction-associated steatotic liver disease (MASLD), including improved overall survival, reduced cardiovascular events, and sustained hepatic improvement over an 8-year period. These findings are in line with published data showing fibrosis regression by semaglutide [33]. Therefore, the findings potentially highlight semaglutide as a promising pharmacologic intervention in MASLD, especially for patients with coexisting metabolic risk factors such as obesity, type 2 diabetes, and dyslipidemia. Given the complexity and heterogeneity of MASLD, future investigations should aim to identify the patient subgroups most likely to benefit from semaglutide and explore the potential of combination therapies to further enhance clinical outcomes.

While our findings demonstrate significant associations between semaglutide use and improved outcomes in MASLD patients, these results should be interpreted in light of the study’s observational design, residual confounding, and analytical limitations. Therefore, causal relationships cannot be definitively established. Prospective, randomized studies are warranted to confirm these observations.

## Figures and Tables

**Table 1:** (**a**) Baseline demographics and comorbidities (after propensity matching); (**b**) baseline laboratory parameters (after matching); (**c**) baseline medication use.

(a)				
Characteristic	Semaglutide Group (*n* = 19,112)	Non-Semaglutide Group (*n* = 19,112)	*p*-Value	St. Diff.
**Demographics**				
**Age** (years), mean ± SD	51.06 ± 12.79	51.26 ± 13.60	0.13	0.0154
**Female**, **n** (%)	11,506 (60.2%)	11,500 (60.2%)	0.95	0.0006
**Race** (%)				
**- White**	13,167 (68.9%)	13,118 (68.6%)	0.59	0.0055
**- Black**	1718 (8.99%)	1696 (8.87%)	0.69	0.0040
**- Asian**	842 (4.4%)	824 (4.3%)	0.65	0.0046
**BMI** (kg/m^2^), mean ± SD	36.60 ± 6.25	34.89 ± 6.84	<0.001	0.2607
**Comorbidities** (%)				
**- Diabetes mellitus**	11,644 (60.9%)	12,010 (62.8%)	0.00012	0.0394
**- Hypertension**	11,046 (57.8%)	11,027 (57.7%)	0.84	0.0020
**- Ischemic heart disease**	1336 (7.0%)	1291 (6.8%)	0.36	0.0093
**- Cerebrovascular disease**	403 (2.11%)	390 (2.04%)	0.64	0.0048
(**b**)				
**Laboratory Parameter**	**Semaglutide Group (** ***n* = ** **19,112)**	**Non-Semaglutide Group (** ***n* = ** **19,112)**	***p*** **-Value**	**St. Diff.**
**Liver function tests**				
**ALT** (U/L), mean ± SD	47.76 ± 54.48 (*n* = 14,573, 76%)	58.15 ± 144.01 (*n* = 14,273, 74%)	<0.001	0.0955
**AST** (U/L), mean ± SD	35.92 ± 53.02 (*n* = 14,197, 74%)	54.24 ± 332.76 (*n* = 13,944, 73%)	<0.001	0.0769
**ALP** (U/L), mean ± SD	85.62 ± 40.17 (*n* = 14,092, 73%)	89.71 ± 53.52 (*n* = 13,763, 72%)	<0.001	0.0865
**GGT** (U/L), mean ± SD	80.21 ± 121.66 (*n* = 873, 4.6%)	111.70 ± 198.84 (*n* = 819, 4.3%)	<0.001	0.1910
**Total bilirubin** (mg/dL), mean ± SD	0.555 ± 0.436 (*n* = 13,901, 73%)	0.646 ± 1.049 (*n* = 13,616, 71%)	<0.001	0.1139
**Albumin** (g/dL), mean ± SD	4.254 ± 0.413 (*n* = 14,094, 74%)	4.151 ± 0.512 (*n* = 13,723, 72%)	<0.001	0.2217
**Metabolic parameters**				
**HbA1c** (%), mean ± SD	7.132 ± 1.829 (*n* = 12,928, 68%)	7.311 ± 1.915 (*n* = 12,705, 66%)	<0.001	0.0958
**Total cholesterol** (mg/dL), mean ± SD	177.99 ± 46.77 (*n* = 11,682, 61%)	181.52 ± 50.89 (*n* = 11,331, 59%)	<0.001	0.0722
**LDL** (mg/dL), mean ± SD	100.32 ± 37.51 (*n* = 12,008, 63%)	101.43 ± 38.35 (*n* = 11,677, 61%)	0.025	0.0292
**HDL** (mg/dL), mean ± SD	43.05 ± 15.01 (*n* = 12,277, 64%)	42.71 ± 16.05 (*n* = 11,873, 62%)	0.089	0.0219
**Triglycerides** (mg/dL), mean ± SD	185.2 ± 164.9 (*n* = 12,291, 64%)	199.9 ± 283.0 (*n* = 11,909, 62%)	<0.001	0.0635
**Hematologic parameters**				
**Platelet count** (×10^3^/μL), mean ± SD	271.1 ± 73.1 (*n* = 12,483, 65%)	261.5 ± 80.2 (*n* = 12,269, 64%)	<0.001	0.1253
**INR**, mean ± SD	1.037 ± 0.174 (*n* = 1931, 10%)	1.126 ± 0.498 (*n* = 2021, 11%)	<0.001	0.2372
**Other markers**				
**AFP** (ng/mL), mean ± SD	3.556 ± 1.841 (*n* = 117, 0.61%)	3.749 ± 3.292 (*n* = 105, 0.55%)	0.587	0.0721
**Creatinine** (mg/dL), mean ± SD	0.867 ± 1.812 (*n* = 14,754, 77%)	0.876 ± 1.411 (*n* = 14,443, 76%)	0.63	0.0056
(**c**)				
**Medication**	**Semaglutide Group (** ***n* = ** **19,112)**	**Non-Semaglutide Group (** ***n* = ** **19,112)**	***p*** **-Value**	**St. Diff.**
Any glucose-lowering agents (non-GLP-1)	18,656 (97.61%)	18,479 (96.69%)	<0.001	0.056
Lipid-lowering agents (any)	8389 (43.89%)	8321 (43.54%)	0.48	0.007
Low-dose aspirin (anti-platelet)	1468 (7.68%)	1493 (7.81%)	0.63	0.005

Legend (**a**): This table presents the baseline demographic and comorbidity data for the propensity score-matched semaglutide-exposed and semaglutide-unexposed MASLD cohorts. *p*-values > 0.05 and standardized differences (St. Diff.) < 0.3 indicate adequate balance between the groups for each characteristic, suggesting a well-matched cohort. SD—standard deviation; St. Diff.—standardized mean differences. Legend (**b**): This table presents the baseline laboratory parameters for the propensity score-matched semaglutide-exposed and semaglutide-unexposed MASLD cohorts. Lab availability varied; percentages indicate the proportion of patients with at least one measurement in the year before the index. Values represent means ± standard deviations for patients with measurements available. *p*-values from t-test for means, with standardized mean difference (St. Diff.) < 0.3 indicating adequate balance between groups. Legend (**c**): This table presents the baseline medication usage for the propensity score-matched semaglutide-exposed and semaglutide-unexposed MASLD cohorts. The percentages represent the proportion of patients using each medication class at baseline. *p*-values and standardized mean differences (St. Diff.) indicate balance between groups, with St. Diff. <0.3 considered well-balanced.

**Table 2:** (**a**) Impact of semaglutide on clinical outcomes over 1, 5, and 8 years of follow-up; (**b**) impact of semaglutide on continuous laboratory data over 1, 5, and 8 years of follow-up; (**c**) impact of semaglutide on categorical laboratory data over 1, 5, and 8 years of follow-up.

(a)						
Clinical Outcomes	Semaglutide Status	1 Year	5 Years	8 Years	HR (95% CI) Over Study	*p*-Value
**Mortality**	Exposed	0.46%	0.68%	0.68%	0.37 (0.30–0.45)	<0.001
	Unexposed	1.57%	3.07%	3.53%		
	*p*-value	<0.001	<0.001	<0.001		
**Survival**	Exposed	99.34%	98.00%	97.99%	--	--
	Unexposed	98.15%	94.43%	90.82%		
	*p*-value	<0.001	<0.001	<0.001		
**Cardiovascular events**	Exposed	3.99%	5.09%	5.10%	0.56 (0.52–0.61)	<0.001
	Unexposed	5.21%	9.02%	9.75%		
	*p*-value	<0.001	<0.001	<0.001		
**Advanced liver disease (any)**	Exposed	4.03%	5.38%	5.39%	0.40 (0.38–0.43)	<0.001
	Unexposed	8.87%	13.28%	14.15%		
	*p*-value	<0.001	<0.001	<0.001		
**ALD (clinical)**	Exposed	3.20%	4.30%	4.35%	0.38 (0.35–0.42)	<0.001
	Unexposed	8.40%	11.30%	12.10%		
	*p*-value	<0.001	<0.001	<0.001		
**ALD (lab)**	Exposed	2.70%	3.60%	3.65%	0.36 (0.33–0.40)	<0.001
	Unexposed	7.10%	10.20%	10.90%		
	*p*-value	<0.001	<0.001	<0.001		
**ALD (medications)**	Exposed	1.60%	2.15%	2.20%	0.32 (0.28–0.36)	<0.001
	Unexposed	5.20%	6.80%	7.10%		
	*p*-value	<0.001	<0.001	<0.001		
**Liver enzyme abnormalities**	Exposed	33.41%	39.20%	39.30%	0.78 (0.76–0.81)	<0.001
	Unexposed	39.42%	47.40%	47.50%		
	*p*-value	<0.001	<0.001	<0.001		
**Synthetic liver markers**	Exposed	1.40%	1.90%	1.95%	0.22 (0.20–0.25)	<0.001
	Unexposed	6.00%	7.30%	8.90%		
	*p*-value	<0.001	<0.001	<0.001		
**Liver transplantation**	Exposed	0.05%	0.05%	0.05%	1.00 (0.40–2.50)	1.000
	Unexposed	0.05%	0.05%	0.05%		
	*p*-value	1.000	1.000	1.000		
**Hepatocellular carcinoma**	Exposed	0.08%	0.09%	0.09%	0.75 (0.40–1.40)	0.370
	Unexposed	0.09%	0.15%	0.15%		
	*p*-value	0.860	0.077	0.077		
**Ascites**	Exposed	0.33%	0.45%	0.45%	0.34 (0.27–0.44)	<0.001
	Unexposed	0.87%	1.31%	1.38%		
	*p*-value	<0.001	<0.001	<0.001		
**Encephalopathy**	Exposed	0.27%	0.37%	0.37%	0.39 (0.30–0.51)	<0.001
	Unexposed	0.58%	0.91%	1.02%		
	*p*-value	<0.001	<0.001	<0.001		
**Varices**	Exposed	0.21%	0.29%	0.29%	0.70 (0.50–0.99)	0.046
	Unexposed	0.27%	0.41%	0.42%		
	*p*-value	0.250	0.045	0.031		
**Ammonia-lowering agents**	Exposed	1.29%	1.95%	1.96%	0.39 (0.35–0.44)	<0.001
	Unexposed	5.40%	4.26%	4.64%		
	*p*-value	<0.001	<0.001	<0.001		
**Diuretics**	Exposed	2.30%	6.00%	6.00%	0.60 (0.55–0.65)	<0.001
	Unexposed	4.80%	10.20%	10.90%		
	*p*-value	<0.001	<0.001	<0.001		
**NSBB**	Exposed	0.47%	1.45%	1.45%	0.35 (0.30–0.41)	<0.001
	Unexposed	0.87%	4.26%	4.64%		
	*p*-value	0.002	<0.001	<0.001		
(**b**)						
**Laboratory Parameter**	**Semaglutide Status**	**1 Year**	**5 Years**	**8 Years**	***p*** **-Value (8 Years)**	
**ALT (U/L)**	Exposed	38.2 ± 32.5	37.4 ± 69.2	37.4 ± 69.2	0.001	
	Unexposed	46.4 ± 140.7	41.9 ± 128.1	42.1 ± 138.4		
	*p*-value	<0.001	0.001	0.001		
**AST (U/L)**	Exposed	29.9 ± 23.0	30.7 ± 145.5	30.7 ± 145.4	<0.001	
	Unexposed	39.9 ± 197.7	37.6 ± 189.1	39.6 ± 243.2		
	*p*-value	<0.001	0.002	<0.001		
**ALP (U/L)**	Exposed	83.7 ± 42.2	83.7 ± 42.1	83.7 ± 42.1	<0.001	
	Unexposed	88.3 ± 60.9	88.3 ± 60.7	88.9 ± 63.2		
	*p*-value	<0.001	<0.001	<0.001		
**GGT (U/L)**	Exposed	74.0 ± 128.6	74.7 ± 123.7	74.7 ± 123.6	<0.001	
	Unexposed	116.6 ± 222.4	110.2 ± 207.2	107.9 ± 204.9		
	*p*-value	<0.001	<0.001	<0.001		
**Bilirubin (mg/dL)**	Exposed	0.56 ± 0.58	0.57 ± 0.65	0.57 ± 0.65	<0.001	
	Unexposed	0.66 ± 1.48	0.67 ± 1.77	0.68 ± 1.85		
	*p*-value	<0.001	<0.001	<0.001		
**Albumin (g/dL)**	Exposed	4.23 ± 0.46	4.22 ± 0.46	4.22 ± 0.46	<0.001	
	Unexposed	4.07 ± 0.59	4.08 ± 0.57	4.07 ± 0.58		
	*p*-value	<0.001	<0.001	<0.001		
**INR**	Exposed	1.05 ± 0.13	1.07 ± 0.15	1.09 ± 0.18	0.07	
	Unexposed	1.09 ± 0.20	1.12 ± 0.22	1.14 ± 0.26		
	*p*-value	0.045	0.031	0.07		
**Platelets (×10^3^/μL)**	Exposed	273.5 ± 71.8	271.0 ± 69.4	268.7 ± 66.9	<0.001	
	Unexposed	262.4 ± 78.3	256.9 ± 75.6	250.8 ± 74.5		
	*p*-value	<0.001	<0.001	<0.001		
**HbA1c (%)**	Exposed	6.73 ± 1.56	6.68 ± 1.55	6.68 ± 1.55	<0.001	
	Unexposed	7.11 ± 1.70	7.10 ± 1.76	7.08 ± 1.74		
	*p*-value	<0.001	<0.001	<0.001		
**LDL (mg/dL)**	Exposed	92.5 ± 36.1	92.2 ± 36.2	92.2 ± 36.3	0.836	
	Unexposed	94.3 ± 36.9	92.7 ± 37.1	92.3 ± 37.3		
	*p*-value	0.003	0.345	0.836		
**HDL (mg/dL)**	Exposed	42.9 ± 15.2	43.5 ± 15.0	43.5 ± 15.0	0.188	
	Unexposed	42.4 ± 16.3	43.6 ± 16.8	43.8 ± 16.9		
	*p*-value	0.047	0.543	0.188		
**Triglycerides (mg/dL)**	Exposed	167.5 ± 154.2	165.2 ± 129.2	165.3 ± 129.2	<0.001	
	Unexposed	183.2 ± 175.3	178.0 ± 169.2	176.2 ± 160.5		
	*p*-value	<0.001	<0.001	<0.001		
**BMI (kg/m^2^)**	Exposed	35.51 ± 6.34	35.28 ± 6.42	35.27 ± 6.42	<0.001	
	Unexposed	34.11 ± 6.64	33.76 ± 6.73	33.71 ± 6.75		
	*p*-value	<0.001	<0.001	<0.001		
(**c**)						
**Categorical Outcome**	**Semaglutide Status**	**1 Year**	**5 Years**	**8 Years**	***p*** **-Value (8 Years)**	
**ALT > 50 U/L**	Exposed	15.36%	18.25%	18.27%	<0.001	
	Unexposed	20.51%	27.56%	28.26%		
	*p*-value	<0.001	<0.001	<0.001		
**AST > 40 U/L**	Exposed	19.6%	22.4%	22.5%	<0.001	
	Unexposed	25.1%	30.8%	32.6%		
	*p*-value	<0.001	<0.001	<0.001		
**Bilirubin ≥ 2 mg/dL**	Exposed	0.63%	0.80%	0.80%	<0.001	
	Unexposed	2.06%	2.61%	2.75%		
	*p*-value	<0.001	<0.001	<0.001		
**Albumin ≤ 2.8 g/dL**	Exposed	0.97%	1.30%	1.30%	<0.001	
	Unexposed	4.23%	5.69%	6.04%		
	*p*-value	<0.001	<0.001	<0.001		
**INR ≥ 1.7**	Exposed	0.21%	0.37%	0.58%	0.002	
	Unexposed	0.54%	0.87%	1.20%		
	*p*-value	0.003	<0.001	0.002		
**Platelets < 150 × 10^3^/μL**	Exposed	8.50%	9.70%	9.80%	<0.001	
	Unexposed	13.30%	15.40%	16.20%		
	*p*-value	<0.001	<0.001	<0.001		
**Platelets < 100 × 10^3^/μL**	Exposed	3.20%	3.60%	3.65%	<0.001	
	Unexposed	5.80%	6.40%	6.70%		
	*p*-value	<0.001	<0.001	<0.001		
**HbA1c > 8.5%**	Exposed	8.22%	10.70%	10.74%	<0.001	
	Unexposed	10.95%	18.00%	18.76%		
	*p*-value	<0.001	<0.001	<0.001		
**Poor metabolic markers**	Exposed	31.13%	36.32%	36.36%	<0.001	
	Unexposed	34.32%	45.80%	46.46%		
	*p*-value	<0.001	<0.001	<0.001		

Legend (**a**): This table summarizes the effects of semaglutide therapy on key clinical outcomes in MASLD patients over 1, 5, and 8 years of follow-up. The outcomes include: mortality, survival, cardiovascular events (defined as cerebrovascular disease, myocardial infarction, or stroke), advanced liver disease (ALD), ALD (clinical) (defined as portal hypertension, ascites, encephalopathy, varices, or liver failure), ALD (lab) (defined as thrombocytopenia, hyperbilirubinemia, hypoalbuminemia), ALD (medications) (defined as the use of lactulose, rifaximin, beta-blockers, or high-dose spironolactone), liver enzyme abnormalities (defined as elevated ALT, AST, ALP, or GGT), synthetic liver markers (defined as hyperbilirubinemia, hypoalbuminemia, or prolonged INR), liver transplantation, hepatocellular carcinoma, ascites, encephalopathy, varices, ammonia-lowering agents (defined as rifaximin or lactulose prescription), diuretics, and NSBB (non-selective beta-blockers). The percentages represent the proportion of patients experiencing each outcome in the semaglutide-exposed and semaglutide-unexposed groups, with associated *p*-values indicating statistical significance. HR = hazard ratio; CI = confidence interval; NSBB = non-selective beta-blockers. Legend (**b**): This table presents the mean values and standard deviations (SD) of various laboratory parameters in MASLD patients with and without semaglutide exposure over 1, 5, and 8 years of follow-up. The laboratory data include: ALT (alanine aminotransferase, U/L), AST (aspartate aminotransferase, U/L), ALP (alkaline phosphatase, U/L), GGT (gamma-glutamyl transferase, U/L), bilirubin (total bilirubin, mg/dL), Albumin (g/dL), INR (international normalized ratio), platelets (cells/μL), HbA1c (hemoglobin A1c, %), LDL (low-density lipoprotein cholesterol, mg/dL), HDL (high-density lipoprotein cholesterol, mg/dL), triglycerides (mg/dL), and BMI (body mass index, kg/m^2^). The mean values and standard deviations are presented for each laboratory parameter in the semaglutide-exposed and semaglutide-unexposed groups at 1, 5, and 8 years of follow-up. The associated *p*-values indicate the statistical significance of the differences between the two groups at each time point. Legend (**c**): This table shows the percentage of MASLD patients meeting specific categorical laboratory criteria in the semaglutide-exposed and semaglutide-unexposed groups over 1, 5, and 8 years of follow-up. The categorical data include: ALT > 50 U/L, AST > 40 U/L, bilirubin ≥ 2 mg/dL, albumin ≤ 2.8 g/dL, INR ≥ 1.7, platelets < 150 × 10^3^/μL, platelets < 100 × 10^3^/μL, HbA1c > 8.5%, and poor metabolic markers (defined as BMI > 35 kg/m^2^, triglycerides > 200 mg/dL, HDL < 40 mg/dL, or HbA1c > 8.5%). The percentages represent the proportion of patients meeting each categorical criterion in the semaglutide-exposed and semaglutide-unexposed groups, with associated *p*-values indicating the statistical significance of the differences between the groups at each time point.

**Table 3:** Number needed to treat to prevent one clinical outcome at different time points.

Outcome	NNT at 1 Year	NNT at 5 Years	NNT at 8 Years
**All-cause mortality**	83	38	28
**Advanced liver disease**	20	17	14
**Cardiovascular event**	71	56	43
**Clinical ALD manifestations**	19	16	14
**Laboratory-defined ALD**	23	19	16
**ALD requiring medications**	28	23	19
**Ascites**	143	111	83
**Encephalopathy**	167	143	111
**Varices**	167	125	100

Legend: This table shows the number needed to treat (NNT) with semaglutide to prevent one clinical event at different time points. NNT was calculated as the reciprocal of the absolute risk reduction. Lower NNT values indicate greater clinical benefit. ALD = advanced liver disease.

## Data Availability

The de-identified electronic-health-record dataset is proprietary to TriNetX and can be accessed by qualified researchers under a data-use agreement with TriNetX (https://trinetx.com).

## Appendix A

**Table A1:** TriNetX definitions and codes of the inclusion and exclusion criteria in the study groups.

Group	Cohorts	Criteria	Codes
**Inclusion Criteria**			
**Age**	Both	Between 18 and 80 years	Age (TNX:9074)
**MASLD diagnosis**	Both	Non-alcoholic steatohepatitis or fatty liver	UMLS:ICD10CM:K75.81 (NASH), UMLS:ICD10CM:K76.0 (Fatty liver)
**Exposure to Semaglutide**	Semaglutide-exposed	Prescription for semaglutide	NLM:RXNORM:1991302 (semaglutide)
	Semaglutide-unexposed	No prescription for semaglutide	
**Exclusion criteria**			
**Advanced liver disease**	Both	No anticoagulants, esophageal varices, ascites, encephalopathy, medications for liver complications, hepatomegaly, liver fibrosis/cirrhosis, or abnormal laboratory values prior to index date	ANTICOAGULANTS: NLM:VA:BL110, Esophageal varices: UMLS:ICD10CM:I85, Ascites: UMLS:ICD10CM:R18, Encephalopathy: UMLS:ICD10CM:G93.40, Medications: neomycin (NLM:RXNORM:7299), rifaximin (NLM:RXNORM:35619), propranolol (NLM:RXNORM:8787), spironolactone (NLM:RXNORM:9997), carvedilol (NLM:RXNORM:20352), Hepatomegaly: UMLS:ICD10CM:R16, Fibrosis/cirrhosis: UMLS:ICD10CM:K74, UMLS:ICD10CM:K74.60, Low platelets: TNX:9020 (≤100 × 10^3^/uL), High bilirubin: TNX:9050 (≥2 mg/dL)
**Liver cancer/transplantation**	Both	No liver transplantation procedures, liver cell carcinoma, or liver transplant status	Liver Transplantation: UMLS:CPT:1007811, Liver cell carcinoma: UMLS:ICD10CM:C22.0, Transplant status: UMLS:ICD10CM:Z94.4
**Viral hepatitis**	Both	No viral hepatitis	Hepatitis B: UMLS:ICD10CM:B19.1, UMLS:ICD10CM:B18.1, Hepatitis C: UMLS:ICD10CM:B19.2, UMLS:ICD10CM:B18.2
**Other liver etiologies**	Both	No other causes of liver disease	Primary sclerosing cholangitis: UMLS:ICD10CM:K83.01, Primary biliary cirrhosis: UMLS:ICD10CM:K74.3, Hemochromatosis: UMLS:ICD10CM:E83.11, Portal vein thrombosis: UMLS:ICD10CM:I81, Wilson’s disease: UMLS:ICD10CM:E83.01, Budd-Chiari syndrome: UMLS:ICD10CM:I82.0, Autoimmune hepatitis: UMLS:ICD10CM:K75.4, Toxic liver disease: UMLS:ICD10CM:K71, Alcoholic liver disease: UMLS:ICD10CM:K70
**Abnormal labs/complications**	Both	No elevated ammonia, abnormal liver function, or portal complications	Ammonia: TNX:LG4629-4 (≥50 umol/L), Liver conditions: UMLS:ICD10CM:K76.1, UMLS:ICD10CM:K76.5, UMLS:ICD10CM:K76.6, UMLS:ICD10CM:K76.7, UMLS:ICD10CM:K76.81, UMLS:ICD10CM:K76.82, Gastric varices: UMLS:ICD10CM:I86.4, Gilbert syndrome: UMLS:ICD10CM:E80.4, Biliary tract diseases: UMLS:ICD10CM:K83, INR: TNX:9032 (≥1.70)

Legend: MASLD—metabolic dysfunction-associated steatotic liver disease; NASH—non-alcoholic steatohepatitis.

## Appendix B

**Table A2:** TriNetX definitions for used terms and codes of clinical outcomes.

Outcome	Definition	Code(s)
**Mortality**	Deceased status	Deceased
**CV events**	Cerebrovascular disease, myocardial infarction, heart failure, or atrial fibrillation/flutter	Cerebrovascular: ICD10:I60-I69, Myocardial infarction: ICD10:I21-I24, Heart failure: ICD10:I50, Atrial fibrillation/flutter: ICD10:I48
**Liver transplantation**	Liver transplant status or procedure	Status: ICD10:Z94.4, Procedure: CPT:1007811, Procedure: ICD10PCS:0FY0
**HCC**	Hepatocellular carcinoma diagnosis	ICD10:C22.0
**Ascites**	Ascites diagnosis	ICD10:R18
**Encephalopathy**	Hepatic encephalopathy diagnosis	ICD10:G93.40, ICD10:K76.82
**Varices**	Esophageal or gastric varices diagnosis	Esophageal: ICD10:I85, Gastric: ICD10:I86.4
**Ammonia-lowering agents**	Rifaximin, lactulose, or neomycin prescription	Rifaximin: RXNORM:35619, Lactulose: RXNORM:6218, Neomycin: RXNORM:7299
**Diuretics**	Furosemide or spironolactone prescription	Furosemide: RXNORM:4603, Spironolactone: RXNORM:9997
**NSBB**	Carvedilol or propranolol prescription	Carvedilol: RXNORM:20352, Propranolol: RXNORM:8787
**Liver inflammatory markers**	Elevated ALT, AST, ALP, or GGT	ALT ≥ 50 U/L: TNX:9044, AST ≥ 50 U/L: TNX:9047, ALP ≥ 80 U/L: TNX:9046, GGT ≥ 80 U/L: TNX:9051
**Synthetic impairment**	Hyperbilirubinemia, hypoalbuminemia or prolonged INR	Bilirubin ≥ 2 mg/dL: TNX:9050, Albumin ≤ 2.8 g/dL: TNX:9045, INR ≥ 1.7: TNX:9032
**Cirrhosis (decompensated)**	Ascites, hepatic encephalopathy, hepatorenal syndrome, spontaneous bacterial peritonitis, thrombocytopenia, hyperbilirubinemia, hypoalbuminemia	Ascites: ICD10:R18, Encephalopathy: ICD10:G93.40, Hepatorenal Syndrome: ICD10:K76.7, SBP: ICD10:K65.2, Platelets ≤ 100 × 10^3^/uL: TNX:9020, Bilirubin ≥ 2 mg/dL: TNX:9050, Albumin ≤ 2.8 g/dL: TNX:9045
**Cirrhosis (compensated)**	Portal hypertension, varices without decompensation	Portal Hypertension: ICD10:K76.6, Esophageal Varices: ICD10:I85, Gastric Varices: ICD10:I86.4, Carvedilol: RXNORM:20352, Propranolol: RXNORM:8787, Cirrhosis: ICD10:K74.6
**ALD (clinical)**	Portal hypertension, ascites, encephalopathy, hepatorenal syndrome, hepatopulmonary syndrome, SBP, varices, liver failure, or cirrhosis	Portal hypertension: ICD10:K76.6, Ascites: ICD10:R18, Encephalopathy: ICD10:G93.40, Hepatorenal syndrome: ICD10:K76.7, Hepatopulmonary syndrome: ICD10:K76.81, SBP: ICD10:K65.2, Varices: ICD10:I85, I86.4, Liver failure: ICD10:K72.9, Cirrhosis: ICD10:K74.6
**ALD (lab)**	Thrombocytopenia, hyperbilirubinemia, hypoalbuminemia, or hyperammonemia	Platelets: TNX:9020 ≤ 100 × 10^3^/uL, Bilirubin: TNX:9050 ≥ 2 mg/dL, Albumin: TNX:9045 ≤ 2.8 g/dL, Ammonia: TNX:LG4629-4 ≥ 50 umol/L
**ALD (medications)**	Neomycin, rifaximin, propranolol, carvedilol, or high-dose spironolactone prescription	Neomycin: RXNORM:7299, Rifaximin: RXNORM:35619, Propranolol: RXNORM:8787, Carvedilol: RXNORM:20352, Spironolactone 100mg: RXNORM:9997

Legend: CV—cardiovascular; HCC—hepatocellular carcinoma; NSBB—non-selective beta-blockers; ALT—alanine aminotransferase; AST—aspartate aminotransferase; ALP—alkaline phosphatase; GGT—gamma-glutamyl transferase; INR—international normalized ratio; SBP—spontaneous bacterial peritonitis; ALD—advanced liver disease.

## Appendix C

**Table A3:** TriNetX definitions for used terms and codes of laboratory outcomes.

Outcome	Definition	Code(s)
**ALT**	Alanine aminotransferase	TNX:9044 [U/L]
**ALT > 50**	Alanine aminotransferase ≥ 50	TNX:9044 ≥ 50 U/L
**AST**	Aspartate aminotransferase	TNX:9047 [U/L]
**ALP**	Alkaline phosphatase	TNX:9046 [U/L]
**GGT**	Gamma-glutamyl transferase	TNX:9051 [U/L]
**Bilirubin**	Total bilirubin [mg/dL]	TNX:9050 [mg/dL]
**Bilirubin > 2**	Total bilirubin ≥ 2 mg/dL	TNX:9050 ≥ 2 mg/dL
**Albumin**	Albumin	TNX:9045 [g/dL]
**Albumin < 2.8**	Albumin ≤ 2.8 g/dL	TNX:9045 ≤ 2.8 g/dL
**INR**	INR	TNX:9032
**INR > 1.7**	INR ≥ 1.7	TNX:9032 ≥ 1.7
**Platelets**	Platelets	TNX:9020 [10^3^/uL]
**Platelets < 150**	Platelets ≤ 150 ×10^3^/uL	TNX:9020 ≤ 150 × 10^3^/uL
**Platelets < 100**	Platelets ≤ 100 ×10^3^/uL	TNX:9020 ≤ 100 × 10^3^/uL
**Ammonia**	Blood ammonia	TNX:LG4629-4 [umol/L]
**Ammonia > 50**	Blood ammonia ≥ 50 umol/L	TNX:LG4629-4 ≥ 50 umol/L
**Creatinine**	Serum creatinine	TNX:9024 [mg/dL]
**BMI**	BMI	TNX:9083
**Total cholesterol**	Total cholesterol	TNX:9000 [mg/dL]
**LDL**	LDL cholesterol	TNX:9002 [mg/dL]
**HDL**	HDL cholesterol	TNX:9001 [mg/dL]
**Triglycerides**	Triglycerides	TNX:9004 [mg/dL]
**Hemoglobin A1c**	Hemoglobin A1c	TNX:9037 [%]
**Hemoglobin A1c > 8.5**	Hemoglobin A1c ≥ 8.5%	TNX:9037 ≥ 8.5%
**AFP**	Alpha fetoprotein	TNX:LG5597-2 [IU/mL]
**Poor metabolic markers**	Elevated LDL, low HDL, elevated triglycerides, or elevated hemoglobin A1c	LDL ≥ 130 mg/dL: TNX:9002, HDL ≤ 40 mg/dL: TNX:9001, Triglycerides ≥ 200 mg/dL: TNX:9004, Hemoglobin A1c ≥ 8%: TNX:9037

Legend: ALT—alanine aminotransferase; AST—aspartate aminotransferase; ALP—alkaline phosphatase; GGT—gamma-glutamyl transferase; INR—international normalized ratio; LDL—low-density lipoprotein; HDL—high-density lipoprotein; AFP—alpha-fetoprotein.
